# Contributions of Resin Cast Etching to Visualising the Osteocyte Lacuno-Canalicular Network Architecture in Bone Biology and Tissue Engineering

**DOI:** 10.1007/s00223-022-01058-9

**Published:** 2023-01-07

**Authors:** Mari Sato, Furqan A. Shah

**Affiliations:** 1 Oral Biochemistry and Molecular Biology, Graduate School of Dental Medicine, Hokkaido University, Sapporo, Japan; 2 Department of Biomaterials, Sahlgrenska Academy, University of Gothenburg, Gothenburg, Sweden

**Keywords:** Bone, Osteocyte, Lacuno-canalicular network, Scanning electron microscopy, Resin cast etching

## Abstract

Recent years have witnessed an evolution of imaging technologies towards sophisticated approaches for visualising cells within their natural environment(s) and for investigating their interactions with other cells, with adjacent anatomical structures, and with implanted biomaterials. Resin cast etching (RCE) is an uncomplicated technique involving sequential acid etching and alkali digestion of resin embedded bone to observe the osteocyte lacuno-canalicular network using scanning electron microscopy. This review summarises the applicability of RCE to bone and the bone-implant interface. Quantitative parameters such as osteocyte size, osteocyte density, and number of canaliculi per osteocyte, and qualitative metrics including osteocyte shape, disturbances in the arrangement of osteocytes and canaliculi, and physical communication between osteocytes and implant surfaces can be investigated. Ageing, osteoporosis, long-term immobilisation, spinal cord injury, osteoarthritis, irradiation, and chronic kidney disease have been shown to impact osteocyte lacuno-canalicular network morphology. In addition to titanium, calcium phosphates, and bioactive glass, observation of direct connectivity between osteocytes and cobalt chromium provides new insights into the osseointegration potential of materials conventionally viewed as non-osseointegrating. Other applications include in vivo and in vitro testing of polymer-based tissue engineering scaffolds and tissue-engineered ossicles, validation of ectopic osteochondral defect models, ex vivo organ culture of whole bones, and observing the effects of gene dysfunction/deletion on the osteocyte lacuno-canalicular network. Without additional contrast staining, any resin embedded specimen (including clinical biopsies) can be used for RCE. The multitude of applications described here attest to the versatility of RCE for routine use within correlative analytical workflows, particularly in biomaterials science.

## Osteocytes—the Master Orchestrators within Bone

The osteocyte lacuno-canalicular network (Ot.LCN) represents an important structural and functional component of bone [1], where the Ot.LCN architecture is closely related to bone material quality [2] and the mechanoresponsiveness of bone [3]. Not only do the osteocytes (Ot) represent > 95% of all the cells in bone [4], evidence from both in vitro and in vivo studies suggests that the osteocyte cytoskeleton, dendritic processes, integrin-based focal adhesions, connexin-based intercellular junctions, primary cilium, ion channels, and extracellular matrix are the major mechanosensors in osteocytes [5]. From each osteocyte, extensive numbers of cellular processes (also referred to as *dendritic projections* or *dendrites*) connect to the bone surface and the marrow space [6–9]. As many as ~ 42 billion osteocytes reside within the average adult human skeleton, and the total number of osteocyte dendritic projections is estimated to be ~ 3.7 trillion [10], which translates to about 88 dendrites per osteocyte. This lacuno-canalicular network (LCN) contains canalicular fluid, and facilitates the transport of various biomolecules (including small proteins) to and from the bloodstream [11], and is considered a key morphological component of osteocyte functionality where osteocytes communicate through gap junctions at the tips of the dendrites [12]. As mechanosensory cells, the osteocytes can sense shear forces caused by fluid flow through the pericellular space [13]. In order to maintain the connectivity of the Ot.LCN across the reversal front, during the remodelling process, the osteocytes can connect their existing dendrites with those of newly embedded osteocytes [6]. Anatomical evidence that the dendrites are in direct contact with the vasculature lends support to the notion that, independently of bone mass, the vascularity of the skeleton and thereby the Ot.LCN fluid volume are key determinants of bone strength [14]. Where the osteocytes play an important role in fracture healing [15], the precise architecture of the Ot.LCN varies with age, disease, and bone type (e.g., cortical or trabecular bone) [16]. Architectural and functional adaptations of the Ot.LCN are also witnessed around implanted biomaterials [17].

## Resin Cast Etching and Scanning Electron Microscopy for Direct Visualisation of the Osteocyte Lacuno-Canalicular Network in Resin Embedded Bone

**Fig. 1:**
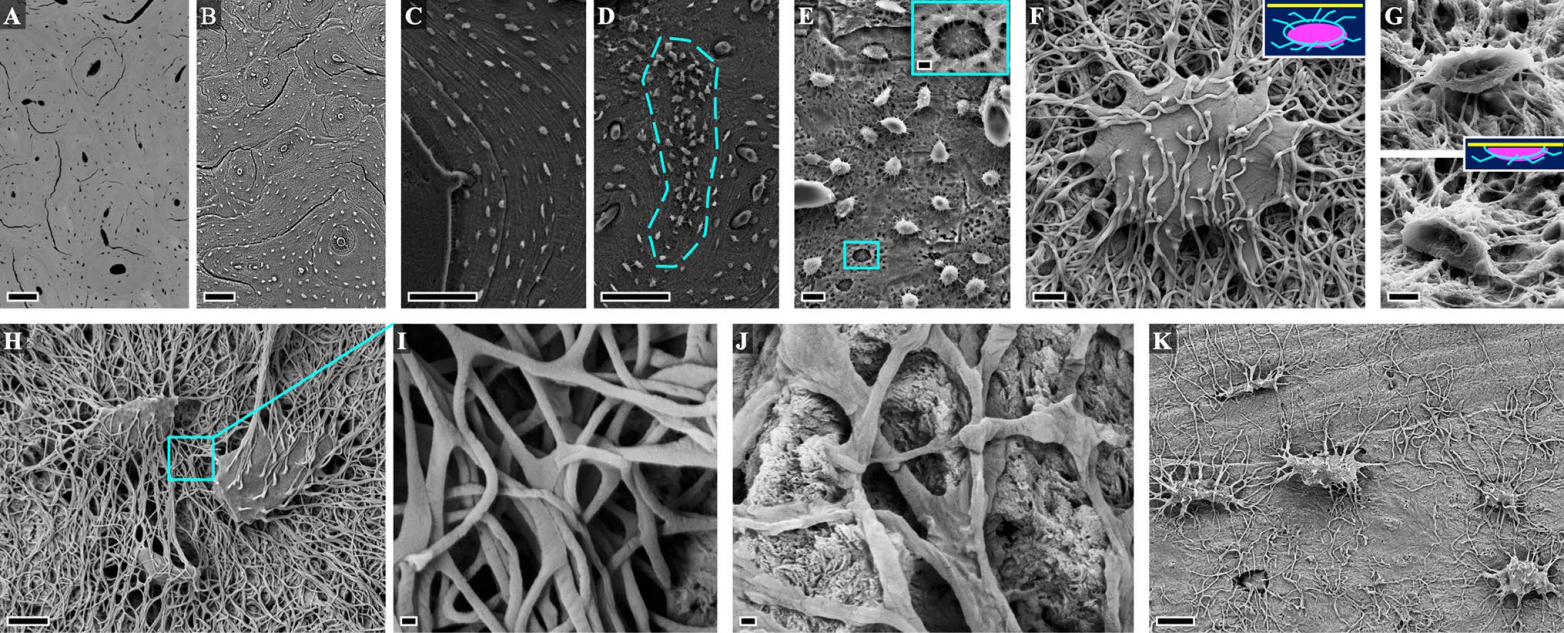
Resin cast etching of bone. **A** Osteons in sheep cortical bone without resin cast etching (BSE-SEM). **B** Osteons in sheep cortical bone after resin cast etching. **C** Lamellar bone. **D** Woven bone (indicated by the broken line). **E** Osteocyte lacuno-canalicular network in rabbit cortical bone. Inset: An incompletely exposed osteocyte within a lacuna. Scale bar = 2 µm. **F** An osteocyte that was completely below the specimen surface before etching and therefore appears intact after etching. **G** Osteocytes that were already exposed at the specimen surface before etching and therefore appear partially cut after etching. Insets in F and G: Illustrations indicate osteocyte location in relation to the specimen surface (yellow line) prior to resin cast etching. **H**,**I** Osteocyte lacuno-canalicular network in rat bone. **J** Canalicular interconnectivity in human alveolar bone. **K** Osteocyte lacuno-canalicular network in human metacarpal bone. Scale bars in A, B, C, and D = 100 µm, E and K = 10 µm, F and G = 2 µm, H = 5 µm, I and J = 200 nm

The Ot.LCN can be imaged with a diverse range of imaging techniques [18]. Variously referred to as *acid-etched resin cast(ing)* or simply *acid-etching*, resin cast etching (RCE) is a sample preparation technique to uncover the Ot.LCN within a limited depth/volume at the surface of a resin embedded specimen for direct visualisation using scanning electron microscopy (SEM) (Fig. 1) [19, 20]. By means of RCE, Curtis and co-workers (in 1985) reported a continuous LCN between osteocytes (described as ‘*flattened oval discs aligned parallel to the lamellae’*) and the vasculature, extending the applicability of this technique to mineralised tissues other than dentine [21]. Preparation typically encompasses rendering the specimen flat by polishing (or alternatively using a thin section of undemineralised bone). The specimen is sequentially exposed to a mild acid, e.g., orthophosphoric acid (H_3_PO_4_) to remove the inorganic phase followed by digestion of the organic phase with sodium hypochlorite (NaOCl). Nevertheless, early investigators have also used hydrochloric acid (HCl) [22] rather than H_3_PO_4_.

The precise protocol requires optimisation due to interspecies differences in bone material properties. As the critical first step, however, the use of lower concentration acid (9% H_3_PO_4_) for a longer exposure time (~ 20–30 s) provides better control than application of higher concentration acid (37% H_3_PO_4_) for a shorter exposure time (~ 3–4 s) [19, 23]. In the second step, 5-min exposure to ~ 5% NaOCl affords an acceptable outcome. The specimen is gently rinsed in deionised water after each step, and finally allowed to dry at ambient conditions for several hours. Depending on the eventual imaging conditions, an electrically conductive coating (e.g., carbon, gold, gold–palladium, platinum, silver, etc.) may be necessary for SEM imaging. The quality of information obtained from the resin cast eventually depends on sample preparation. While a minor role is played by shrinkage artefacts related to fixation and dehydration [24], poor infiltration and polymerisation of the embedding resin may lead to artefacts such as areas that appear partially or completely devoid of Ot.LCN. This scenario can be particularly troublesome in the case of specimens that are too large or inhomogeneously dense to allow unimpeded flow of the embedding medium.

Using RCE, Okada and co-workers demonstrate variations in arrangement of the Ot.LCN between alveolar and basal areas of rat mandibles [25]. Progressing beyond qualitative descriptions of osteocyte shape, canalicular density, and overall vastness of the LCN, Pazzaglia and Congiu classified canaliculi into equatorial and radial types and established elegant quantitative metrics of Ot.LCN morphology, including osteocyte (or lacunar) density per unit surface area (#/mm^2^), lacunar surface area (µm^2^), number of canaliculi per osteocyte lacuna or dendricity (#/Ot.Lc), inter-lacunar canalicular length (µm), and canalicular diameter (nm) [26]. The osteocyte density is typically taken as the number of osteocytes or osteocyte lacunae per unit area of the mineralised compartment (N.Ot/B.Ar), but infrequently also taken as per total area, i.e., taking into consideration both the mineralised and unmineralised tissue compartments (N.Ot/Tt.Ar) [27]. Certain osteonal bone-specific metrics have also been defined. These include: (*i*) indirect canalicular connectivity, i.e., presence of connections between the radial canaliculi of an osteocyte and the transverse canaliculi of another osteocyte from a different lamella of the same osteon (%) [28], and (*ii*) intra-osteon maximum canalicular length, i.e., the longest visible canaliculus reaching the outermost lamella or a peripheral lacuna of the specific osteon (µm) [28, 29]. The information obtained by RCE can be further enriched by correlative backscattered electron scanning electron microscopy (BSE-SEM) [28, 30].

Compared to BSE-SEM, where only the osteocytes at the specimen surface can be seen, 2D imaging of the same field of view following RCE will reveal osteocytes in greater numbers, i.e., higher osteocyte density. The etching depth can vary as a function of reagent concentration and application time. With respect to the surface of a polished specimen (i.e., before RCE), the physical appearance of osteocytes located at the surface differs considerably from those present a few micrometres below the surface. Osteocytes at the specimen surface (seen by BSE-SEM) are partially cut and have lost some portion of the cell. On the contrary, sub-surface osteocytes (not seen by BSE-SEM) appear intact after RCE, since the entire cell becomes exposed, and are ideal candidates for quantitative measurements (e.g., dendricity).

## Changes in Osteocytes Due to Ageing and Disease

The architecture of the Ot.LCN is altered in several conditions including ageing, osteoporosis, skeletal unloading, spinal cord injury, osteoarthritis, irradiation, and high calcium demand, and many of these changes have been investigated using RCE (Table 1).

**Table 1:** Effect(s) of ageing and disease on the osteocyte lacuno-canalicular network

Condition	Species	Effect(s) on Ot.LCN	Refs
Ageing	Rat	Age-dependent change in osteocyte shape (from round to ellipsoidal and eventually flat) and decrease in canalicular interconnectivity/complexity	[25]
Natural and artificial ageing	European eel	Decreased osteocyte density in both natural and pharmacologically induced ageing	[31]
Ageing	Human	Age-dependent decreases in dendricity, indirect canalicular connectivity, and maximum canalicular length	[28]
Ageing	Human	Age-dependent decreases in dendricity and maximum canalicular length	[29]
Ageing	Human	Age-dependent decrease in dendricity	[32]
Osteoporosis	Rat (± OVX)	Decreased osteocyte density and dendricity due to OVX. Changes are reversed by Scl-Ab	[33]
Osteoporosis	Rat (± OVX)	No effect of hPTH, ALN, or RLX demonstrated	[35]
Osteoporosis	Rat (± OVX)	Regular osteocyte shape, higher dendricity, and branched canaliculi under stimulation with low-magnitude high-frequency vibration during fracture healing	[36]
Postmenopausal osteoporosis	Human	No change in Ot.LCN morphology in osteoporotic vs. age-matched healthy individuals	[29]
Skeletal unloading	Human	Greater loss of dendricity in long-term immobilised vs. age-matched osteoporotic and healthy individuals	[29]
Hindlimb suspension	Rat (± OVX)	Decreased osteocyte density and dendricity in normal/healthy and OVX animals. Changes are reversed by Scl-Ab	[33]
Spinal cord transection	Rat	Lower dendricity and osteocyte density per total area, deformed and disorganised osteocytes. Changes are reversed by Scl-Ab	[27]
Spinal cord transection	Rat	Lower dendricity. Changes are reversed by 15 Hz pulsed electromagnetic fields	[38]
Osteocytic osteolysis	Mouse (Ctsk-Cre/R26R)	Reversibly increased osteocyte lacunar area and canalicular diameter during high calcium demand	[39, 40]
Osteoarthritis	Human	Deformed, irregular-shaped osteocytes with fewer canaliculi	[41]
Irradiation	Dog	Decreased osteocyte density, dendricity, and canalicular connectivity between osteocytes and periosteum. Wider Haversian canals	[42]
Irradiation and implant healing	Dog	Narrower Haversian canals	[43]

### Ageing

Okada and co-workers have reported distinct differences in Ot.LCN morphology between juvenile (3-week-old), adult (12-week-old), and aged (80-week-old) rats [25]. In juvenile rats, the osteocytes appear round while the canaliculi are short and thick. In adult rats, the osteocytes are ellipsoidal, with greater canalicular interconnectivity, as the canaliculi branch, twist, and fuse with neighbouring canaliculi to form an intricate network. Notably, the number of canaliculi going from an osteocyte to the central vascular canal is greater than the number of canaliculi going outward from the same lacuna to other neighbouring osteocytes. In aged rats, the osteocytes are slender and flat, with fewer canalicular communications, and a comparatively simpler canalicular network. In marine species such as the European eel (*Anguilla anguilla*), the osteocyte density declines from ~ 288 ± 110 mm^−2^ at the yellow (juvenile) eel stage, which lasts for 5–20 years, to ~ 201 ± 50 mm^−2^ at the silver (adult) eel stage. Artificial maturation can be accomplished through administration of human chorionic gonadotropin to male eels while carp pituitary extract and 17,20β-dihydroxy-4-pregnen-3-one to female eels. Such pharmacologically induced ageing results in the osteocyte density decreasing to ~ 134 ± 46 mm^−2^ prior to spawning towards the end of the silver eel stage [31].

Age-dependent variation in Ot.LCN interconnectivity is also reported in the human femoral cortex. In a cohort of young (20–40 y) and aged (70–95 y) individuals, the incidence of indirect canalicular connectivity is ~ 25% lower and the maximum canalicular length is 27% reduced in aged individuals. Furthermore, dendricity decreases by ~ 28%, from ~ 22 in young individuals to ~ 16 in aged individuals [28]. Similar trends have been reported in a cohort of young (34 ± 7 y) and aged (81 ± 7 y) individuals where dendricity decreases by ~ 12.4%, from ~ 19.4 in young individuals to ~ 17 in aged individuals, while the maximum canalicular length is ~ 37% reduced, from ~ 72 µm in young individuals to ~ 46 µm in aged individuals [29]. Similar age-related patterns in dendricity have also been reported in human auditory ossicles [32].

### Osteoporosis, Skeletal Unloading, and Spinal Cord Injury

Morphological abnormalities of the Ot.LCN such as decreased osteocyte density and reduced dendricity are observed under experimentally induced osteoporosis via ovariectomy (OVX) in rats. These alterations are reversed by the administration of sclerostin monoclonal antibody (Scl-Ab) [33] and blockade of the protein sclerostin, which is an osteocyte-secreted Wnt signalling antagonist and inhibitor of bone formation [34]. Changes in local nanomechanical properties arising from different sequential treatment regimens of various anti-osteoporotic agents, e.g., human parathyroid hormone (hPTH), alendronate (ALN), raloxifene (RLX), have been correlated with osteocyte shape descriptors (i.e., major and minor axes) and osteocyte density in healthy and OVX rats. However, none of the therapeutic regimens led to demonstrable effects on the extracellular matrix nor did Ot.LCN morphology vary independently of extracellular matrix nanomechanical properties in OVX rats [35]. Stimulation with low-magnitude high-frequency vibration is associated with more regular osteocyte shape, increased dendricity, and densely branched canaliculi in both healthy and OVX rats from approximately one week through to six weeks post-fracture [36].

Related to increased sclerostin production by osteocytes due to skeletal unloading (i.e., disuse osteoporosis) [37], long-term immobilisation causes a specific pattern of cortical bone and osteocyte deterioration different from postmenopausal osteoporosis. In human femoral cortices of long-term immobilised, age-matched osteoporotic, and age-matched healthy females, dendricity in long-term immobilised individuals (~ 12) is lower than in aged-matched osteoporotic (~ 15) and age-matched healthy (~ 17) individuals. The intra-osteon maximum canalicular length is comparable between long-term immobilised (~ 48.9 µm), aged-matched osteoporotic (~ 52.7 µm), and age-matched healthy (~ 45.6 µm) individuals [29]. Interestingly, no differences in dendricity or maximum canalicular length are observed between postmenopausal osteoporosis and age-matched healthy females [29]. Hindlimb suspension has been used experimentally to simulate long-term immobilisation and disuse osteopenia in rodents. This condition has detrimental effects on osteocyte density and dendricity in both healthy and OVX rats. However, administration of Scl-Ab helps to restore the discrepancy [33].

Spinal cord injury is associated with abnormal neurological function and extreme immobilisation. Insights have been gained into the potential impact of traumatic spinal cord injury induced experimentally by transection of the spinal cord. In rats, the effects of spinal cord injury on the Ot.LCN are evident within 8 weeks [27, 38]. Administration of Scl-Ab once per week [27] and whole-body stimulation with pulsed electromagnetic fields [38] have been shown to be effective in restoring some of the alterations in Ot.LCN morphology following transection of the spinal cord. Transection at the 4th thoracic vertebra leads to ~ 30% lower dendricity and ~ 40% decreased osteocyte density per total area (i.e., including the marrow space), compared to spinal cord-intact animals. Systemic administration of Scl-Ab appears to counteract such alterations. Osteocyte density per bone area (i.e., excluding the marrow space), however, remains unaffected after spinal cord injury, with or without Scl-Ab. One explanation for this discrepancy between osteocyte density *per total area* and osteocyte density *per bone area* is loss of bone mass following spinal cord injury, which is in agreement with decreased bone volume (BV/TV) and increased trabecular spacing (Tb.Sp), as reported. Furthermore, the osteocytes in spinal cord-transected animals are markedly deformed, appearing round with a rough inner lacunar wall, and disorganised, while Scl-Ab treatment maintains the typical spindle shape and the well-ordered arrangement [27]. In a similar model, transection at the 12th thoracic vertebra leads to ~ 41% lower dendricity and consequently impaired dendrite connectivity compared to spinal cord-intact animals. However, 2 h per day exposure to pulsed electromagnetic fields delivered as a pulsed burst at 15 Hz frequency restores the deterioration in dendricity in spinal cord-transected animals [38].

### Osteocytic Osteolysis, Osteoarthritis, and Irradiation

Cathepsin K (Ctsk) is a cysteine proteinase that mediates bone resorption and expressed not only in osteoclasts but also in osteocytes. In Ctsk-Cre/R26R mice, the osteocyte lacunar area increases reversibly in both cortical and trabecular bone during phases of high calcium demand, such as lactation, while a pronounced increase in canalicular diameter is observed only in trabecular bone [39, 40]. Degenerative conditions such as osteoarthritis are also associated with markedly deformed osteocytes having an irregular and rounded appearance and very few canaliculi compared to spindle shaped, well-organised and interconnected osteocytes in bone from individuals without subchondral bone sclerosis or cartilage degeneration [41]. Irradiation also leads to changes in Ot.LCN morphology and the vasculature. In the alveolar bone, the most dramatic change is seen in dendricity, from ~ 17 in nonirradiated bone to ~ 9 in irradiated bone, i.e., a ~ 44% reduction. Likewise, canalicular connectivity between osteocytes and the periosteum is also impaired. And while osteonal diameter is not affected, the Haversian canals in irradiated bone are ~ 28% wider and osteocyte density is reduced by ~ 12%. [42]. Irradiation compromises the healing environment for osseointegrated implants. In previously irradiated alveolar bone, the peri-implant Ot.LCN and vasculature have been shown to be particularly affected. However, following submerged healing of commercially pure titanium (cp-Ti) cylinders to restore masticatory function, the Haversian canals in irradiated bone are found to be ~ 22% narrower [43]. This reduction in Haversian canal diameter is contrary to observations in native bone [42], suggesting differences between how irradiation impacts native bone and how the healing environment might respond post-irradiation.

## The Osteocyte Lacuno-Canalicular Network in Transgenic Animal Models

In several animal models of gene dysfunction, altered osteocyte morphology and organisation of the associated canalicular network have been demonstrated using RCE (Table 2).

**Table 2:** Summary of RCE findings from investigations of gene dysfunction

*Gene* or PROTEIN	Ot density	Canaliculi	Ot Distribution	Ot Size	Ot Shape	LCN	Osteoid	Ot.LCN walls	Refs
*DMP-1*^*†*^	–	Decreased ↓	Clustered; Disorganised	Larger ↑	Deformed	–	More ↑	Rough; Buckled	[44–49]
*Klotho*	–	Decreased ↓	Randomly oriented	–	–	–	–	–	[50]
*DMP-1 and Klotho*	–	Decreased ↓	Disorganised	Larger ↑	–	–	–	–	[50]
*DSPP*	–	Decreased ↓	Irregular	Larger ↑	–	Disorganised; Reduced propagation	–	–	[51]
*DSPP N-terminus overexpression*	–	Decreased ↓	Irregular	–	Deformed	Disorganised	–	–	[52]
Col4a3^*††*^	–	–	–	–	Round	–	–	–	[53]
*Osx*	Low ↓	Decreased ↓	–	–	Deformed	–	–	–	[54]
*BMPR-1A*	High ↑	Decreased ↓	Irregular; Disorganised	Larger ↑	–	–	–	–	[55]
*ERK-1, ERK-2*	–	Decreased ↓	–	–	–	–	–	–	[56]
Nf1	–	Decreased ↓	Irregular; Disorganised	–	Deformed, Round	Disorganised	–	Coarse	[57, 58]
*CSF-1*	Low ↓	Decreased ↓	Disorganised	–			More ↑	Rough	[60]
*FAM20C*	–	Decreased ↓	–	–	Abnormal, Deformed		–	–	[62, 63]
Phex	–	–	–	–		Severely disturbed	More ↑	Buckled	[64]
Vhl	Low ↓	Decreased ↓	Disorganised; Randomly oriented	–	Deformed		–	–	[65]

^†^Transgenic expression of DSPP rescues Ot.LCN defects observed in *Dmp1* knockout mice [48, 49]

^††^Genetic or pharmacological supplementation with DMP1 corrects osteocyte morphological changes [53]

### Dentine Matrix Protein-1, Klotho, and Dentine Sialophosphoprotein

In the absence of dentine matrix protein-1 (DMP-1), e.g., *Dmp1* null mice, the inner lacuno-canalicular wall appears rough and buckled, and fewer canaliculi are present [44, 45]. Furthermore, abnormal lacuno-canalicular system in *Dmp1* knockout bone shows a reduction in the number of canaliculi but enlarged osteocyte lacunae with rough lacunar walls. Re-expression of full-length DMP-1 rescues the skeletal abnormalities of *Dmp1* null mice [46]. There is also evidence of periodontal breakdown in the *Dmp1* null mouse model of hypophosphatemic rickets, where the Ot.LCN is disorganised, lacunae are clustered together, and a large amount of osteoid is present [47]. Expression of dentine sialophosphoprotein (DSPP*)* is markedly reduced in *Dmp1* knockout mice, where the osteocyte lacunae appear larger, and disorganised with fewer canaliculi, both in alveolar bone and in long bones [48, 49]. Transgenic expression of DSPP rescues Ot.LCN defects noted in *Dmp1* knockout mice to varying levels in the alveolar bone [48] and in the femur [49]. Furthermore, in *Dmp1* and Klotho deficient (*Dmp1*^*–/–*^* kl/kl*) mice, osteocytes are poorly organised, visibly larger in size, and exhibit a complete lack of dendritic processes. In Klotho-deficiency, alone, only a moderate reduction in the number of canaliculi and random orientation of osteocytes are observed [50]. In *Dspp* null mice, osteocyte lacunae appear larger and irregularly distributed, along with disorganised and fewer canaliculi that exhibit a markedly reduced propagation into the surrounding bone [51]. Overexpression of the NH_2_-terminal fragment of DSPP further aggravates the periodontal defects of *Dspp* null mice. The spectrum of morphological alterations is similar to *Dspp* null mice, but more intense, where the osteocyte lacunae in the alveolar bone appear irregular with disorganised and fewer canaliculi [52].

### Collagen Alpha-3(IV) Chain

The 129SvJ *Col4a3*^–/–^ and B6 *Col4a3*^–/–^ mouse models lack the collagen alpha-3(IV) chain (Col4a3) and display several features of human chronic kidney disease, including progressive loss of kidney function and alterations in bone and mineral metabolism. Compared to their respective wild-type counterparts, *Col4a3* null mice exhibit round osteocytes. However, genetic or pharmacological supplementation with DMP-1, which plays a key role in terminal differentiation of osteocytes, has been found to correct the morphological alterations in *Col4a3* null mice [53].

### Osterix

The osteoblast-specific transcription factor osterix (Osx) is required for osteoblast differentiation into mature osteoblasts and osteocytes. Postnatal ablation of Osx leads to markedly deformed osteocytes, a decreased number of osteocytes throughout the cortex (close to both periosteum and endosteum, and in the middle of the cortex), and very few canaliculi resulting in a greatly reduced density of the canalicular network [54].

### Bone Morphogenetic Protein Receptor Type 1A

Bone morphogenetic proteins (BMPs) are multi-functional growth factors and are known to regulate bone development and regeneration. Bone morphogenetic protein receptor type 1A (BMPR-1A) is a potent receptor of BMP. Defective osteocyte formation and morphology are observed in conditional knockout mice with deletion of *Bmpr1a*. The osteocytes appear enlarged with a sharp increase in osteocyte density and a poor spatial distribution pattern, and severe reduction in the number of canaliculi [55].

### Extracellular Signal-Regulated Kinase

The extracellular signal-regulated kinase (ERK) mitogen-activated protein kinase (MAPK) pathway plays an essential role in mediating fibroblast growth factor signalling in skeletal cells. In the absence of ERK-1 and ERK-2 in limb mesenchyme of *Prx1-Cre* mice, the osteocytes lack dendritic processes, indicating that ERK-1 and ERK-2 inactivation disrupts the formation of the Ot.LCN [56].

### Neurofibromin 1

Neurofibromin is an important protein for skeletal development and growth. Inactivation of *Nf1* in the mesenchymal lineage of *Nf1Prx1* mice results in severe skeletal pathology in osteoblast-specific conditional knockout mice. In *Nf1Prx1* mice, the osteocytes are irregularly distributed, less elliptical (or more spherical) and their orientation deviates from the expected lamellar direction, and the canalicular network is disorganised. Furthermore, the morphological changes are most pronounced in the proximity of muscle attachment sites, which are areas exposed to high mechanical loads [57]. *Nf1* deletion has also been shown to result in a disorganised distribution of osteocytes in osteocyte-specific conditional knockout mice. Moreover, the canaliculi are disorganised and coarse, and the number of canaliculi per cell is significantly reduced. Overall, *Nf1* deficiency in osteocytes appears to disrupt the mineralisation process, leading to an osteopenic change and osteomalacia-like bone phenotype [58].

### Colony-Stimulating Factor 1

Colony-stimulating factor 1 (CSF-1) is highly expressed by osteoblasts and osteocytes to regulate osteoclast-mediated bone remodelling [59]. Disruption of CSF-1 leads to osteopetrosis and osteocyte defects. RCE shows that *Csf1* knockout osteocyte lacunae are disorganised, decreased in number, and have rough walls. In addition to significant reduction in the overall density of the dendrite network, large amounts of osteoid are present in the extracellular matrix [60].

### Golgi Serine/Threonine Protein Kinase FAM20C

Human FAM20C and mouse DMP4 amino acid sequences are 69% identical (73% similar) [61]. Using conditional knockout mice with mineralised tissue-specific deletion of *Fam20c*, FAM20C has been shown to play a critical role in the development of the vertebra [62, 63]. Here, osteocytes in the vertebrae [62] and tibiae [63] of conditional knockout mice appear to lose their normal morphology and have fewer canaliculi.

### Phosphate-Regulating Neutral Endopeptidase

In global and conditional (osteoblasts and osteocytes) phosphate-regulating neural endopeptidase (*Phex)* knockout mice, the inner lacuno-canalicular wall appears buckled and enlarged, with severely disturbed canalicular organisation in addition to abundant osteoid. Aberrant PHEX function, therefore, leads to a bone phenotype indistinguishable from that of hypophosphatemic (*Hyp)* mice, the murine homolog of human X-linked hypophosphatemia and Vitamin D resistant rickets [64].

### Von Hippel-Lindau Tumour Suppressor

The E3 ubiquitin ligase von Hippel-Lindau targets various proteins for degradation, including hypoxia-inducible factor 1-alpha (HIF-1a), and is expressed in many tissues and cell types. Disruption of the von Hippel-Lindau (*Vhl)* gene in mature osteoblasts/osteocytes in mice activates the HIF-1 signalling pathway in osteocytes, which results in disorganised, randomly oriented, and irregularly contoured osteocytes. The osteocyte density is decreased together with the presence of fewer canaliculi [65].

## Osteocytes in Peri-Implant Bone

**Fig. 2:**
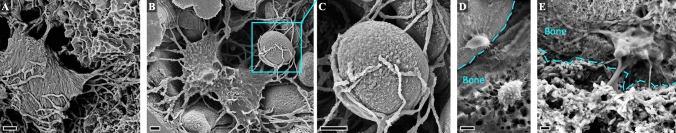
Osteocytes in peri-implant bone. **A** Osteocyte attachment via interdigitation between canaliculi and the topographical features of an acid-etched titanium implant in rat bone. **B**,**C** Canaliculi wrapped around a spherical feature on a selectively laser-ablated titanium implant in rabbit bone. Adapted with permission from Shah et al. 2019. Copyright 2019, Elsevier [66]. **D** Direct connectivity between osteocytes and a 3D printed CoCr implant in rabbit bone (broken line demarcates the bone-implant interface). **E** An osteocyte attached to a multi-component CaP implant in sheep bone (broken line demarcates the bone-implant interface). Scale bars in A, B, and C = 2 µm, D = 5 µm, E = 1 µm

The osteocytes and the associated LCN are fundamental components of bone formed around implant biomaterials [66]. These include load-bearing implants intended for permanent anchorage in bone (typically metals and alloys), degradable biomaterials that stimulate new bone formation and gradually resorb, and tissue engineering scaffolds seeded with various relevant cell types and biological cues (Fig. 2).

### Osseointegrated Metallic Biomaterials

The Ot.LCN in peri-implant bone shows several structural changes related to factors such as healing kinetics and the physico-chemical properties of the implant biomaterial [17]. Presumably, a dense and well-aligned network of dendritic processes in the vicinity of the implant surface may be able to detect compressive and tensile strains imposed on the interfacial tissue, allowing for structural adaptations to maintain homeostasis. Speculatively, close adaptation and attachment of the lacuno-canalicular network to the implant surface may behave as a physical factor contributing towards bone-implant interlocking. Direct attachment of osteocytes to the surface of implants, through dendritic processes, is worth examining [67, 68], and in principle, can be observed as early as mineralised bone is detectable after implantation. When witnessed between osteocytes and materials otherwise considered to integrate poorly, e.g., CoCr [69, 70], such direct connectivity is interpreted as evidence of osseointegration.

RCE has been employed extensively to observe the spatial relationships between the Ot.LCN and bone anchored implants, including topologically modified titanium [71, 72]. Osteocytes are generally aligned with their long axes parallel to the implant surface with long canaliculi extending towards it. Osteocytes form an interconnected lacuno-canalicular system and attach, through canaliculi, to acid-etched [71] and grit-blasted [72] surfaces. Interconnectivity between neighbouring osteocytes and formation of extensive canalicular networks in the vicinity of the implant surface implies cell-to-cell communication. Where implant surface features are of appropriate dimensions, e.g., sub-micron topography achieved through acid-etching, the canaliculi can closely interdigitate with the topographical features [71]. Direct attachment of osteocytes to alumina grit-blasted implants has also been demonstrated in OVX rats [73]. Furthermore, highly interconnected osteocytes are also reported adjacent to nanopatterned implant surfaces characterised by ~ 75 nm wide hemispherical features produced by colloidal lithography [30]. Nanopatterning achieved by alkaline-etching also has a demonstrable positive impact on osteocyte dendricity compared to acid-etched, micro-rough implants in a rat model [74].

Laser-ablation of titanium under ambient conditions generates a 50–200 nm thick surface TiO_2_ layer that is superimposed over micrometre-sized droplets of resolidified metal. Experiments conducted in rabbits show that canaliculi extend several micrometres towards the implant surface [67]. Interestingly, the canaliculi remain attached to the implant surface despite the application of disruptive mechanical forces such as during the acquisition of removal torque measurements. Adjacent to functionally loaded laser-ablated clinical dental implants (i.e., in human), osteocytes are aligned parallel to both the lamellar direction of bone and to the micrometre-scale contour of the implant surface, which points towards an important role of implant surface contour in guiding the eventual microstructure of peri-implant bone. From these osteocytes, canaliculi not only extend towards the implant surface and branch in close proximity to the channel-like surface TiO_2_ layer but also extend in the opposite direction (i.e., away from the implant surface) towards Haversian canals [75].

In newly formed bone adjacent to micro-rough implants [72] and additively manufactured macro-porous Ti6Al4V implants [68], the osteocyte density is typically higher than in the native bone distant from the implant. Higher osteocyte densities are attributable to the presence of relatively younger bone (compared to native bone) and woven bone that is yet to remodel. Tantalum coatings on porous additively manufactured Ti6Al4V fracture-fixation plates also support the attachment of osteocytes through interconnected canaliculi, providing further evidence in favour of the osseointegrative properties of tantalum [76].

At least in small animal models, repetitive mechanical loading of osseointegrated implants has been shown to increase osteocyte density together with changes in osteocyte morphology such as decreased ellipticity and higher dendricity in peri-implant bone [77]. In experimental setups without mechanical stimulation/loading, a disorganised arrangement of osteocytes is noted in areas of new bone adjacent to micro-rough implants compared to areas of native lamellar bone [72]. Similar observations have been made in OVX rats, where adjacent to both minimally rough and micro-rough implant surfaces, the osteocytes exhibit a disorganised arrangement and appear less elliptical than those in native lamellar bone [73].

Osteocyte attachment to highly complex, additively manufactured, macro-porous geometries has also been reported [68, 69]. After long-term submerged healing in sheep, the dendricity adjacent to the surface of additively manufactured, macro-porous and solid Ti6Al4V implants is 38–42% higher than in areas of native lamellar bone [68]. Here also, osteocytes adjacent to the implant surface appear less elliptical, but somewhat larger than those in areas of native lamellar bone [68].

In bone adjacent to functionally loaded, laser-ablated clinical dental implants, the dendricity in peri-implant bone (~ 25) is higher than in the native bone outside the implant thread (~ 17), pointing towards biomechanical differences between the two anatomical locations [75].

### Degradable Biomaterials for Bone Repair

Certain bone repair materials are designed to degrade in vivo. Consequently, the bone-biomaterial interface migrates over time. It has been demonstrated that bioactive glass supports osteocyte attachment via dendritic processes that appear to pass through the calcium phosphate (CaP) rich interfacial layer and reach the silica rich surface of the reacted bioactive glass [78]. A concentric arrangement of the Ot.LCN in bone that is formed in response to multi-component CaP materials, beyond the physical confines of the skeletal envelope, offers evidence for remodelling in extraskeletal bone [79]. Composite beads containing a pullulan–dextran mixture as the organic phase and hydroxy(l)apatite as the inorganic constituent have been investigated in a sinus floor augmentation model in sheep. The presence of a dense network of osteocytes has been reported around the organic–inorganic composite beads with numerous zones of cells in direct contact with the microbeads, at 3 and 6 months, with evidence of neovascularisation and direct contact between osteocytes and blood vessels [80]. Similar observations have been made for clinically used deproteinised bovine bone mineral (Bio-Oss®) in a sinus floor augmentation model in sheep. The bone formed around deproteinised bovine bone mineral particles is characterised by highly aligned networks of osteocytes, and connectivity between osteocytes and neighbouring blood vessels through canaliculi [80].

### Tissue Engineering Scaffolds and Tissue-Engineered Ossicles

3D printed polycaprolactone (PCL) scaffolds with CaP coatings and recombinant human bone morphogenetic protein 7 (rhBMP-7) has been a popular direction in bone tissue engineering. When PCL-CaP constructs seeded with either human mesenchymal stem cells or human primary osteoblasts are loaded with rhBMP-7 and subcutaneously implanted in immunodeficient mice, an abundance of morphologically intact osteocytes with highly interconnected dendritic processes is witnessed by 8 weeks post-implantation. As may be expected, the osteoblasts lead to ~ 79% more osteocytes per unit area than the human primary mesenchymal stem cells [81]. Segmental tibial defects in sheep treated with PCL-CaP constructs loaded with both platelet-rich plasma and rhBMP-7 show mainly woven bone with a disorganised arrangement of osteocytes by three months post-implantation. The canalicular network appears mostly disrupted and damaged. The woven bone is widely replaced by lamellar bone at 12–15 months post-implantation. In addition to the presence of blood vessels and primary osteon formation, osteocytes are found in direct contact with scaffold struts [82]. Human mesenchymal stem cells can also be seeded onto tubular PCL-CaP constructs by sequential static culture followed by dynamic culture in a rotating bioreactor. Loaded with rhBMP-7 and embedded in fibrin glue, such constructs are subcutaneously implanted in immunodeficient mice to obtain tissue-engineered, humanised ossicles. At 10–14 weeks, an abundance of osteocytes with highly interconnected dendritic processes has been interpreted as a functional osteocyte network within the newly formed bone [83, 84].

### Medication-Related Osteonecrosis of the Jaw and BMP-2 Delivery

RCE has been found to be useful in evaluating a model multi-component hydrogel system consisting of star-shaped polyethylene glycol, Arg-Gly-Asp tripeptides, and maleimide functionalised heparin as a delivery mechanism for rhBMP-2. In a rat model of medication-related osteonecrosis of the jaw (MRONJ), induced by zoledronic acid infusion after tooth extraction, controlled release of rhBMP-2 is associated with increased osteocyte density. On the other hand, defects not treated with rhBMP-2 show empty lacunae, attributable to areas of non-vital bone, which is consistent with the expected histological appearance of MRONJ where hematoxylin and eosin staining demonstrate karyorrhexis, karyolysis, and pyknosis [85].

## Unconventional Applications of Resin Cast Etching

RCE has been employed in several unique scenarios. In mouse femurs maintained under ex vivo culture, exposure to transforming growth factor-beta 1 (TGF-β1) increases dendricity while TGF-β1 receptor inhibition reduces dendricity [86]. In tissue engineering using primary human osteoprogenitor cell-laden tubular PCL-CaP constructs, detection of osteocyte-like dendritic cells points towards in vitro osteogenic differentiation of the seeded cells [87]. Likewise, preservation of Ot.LCN architecture in bovine osteochondral plugs supplemented with rhBMP-7 and subcutaneously implanted in rats attests in favour of this setup as an ectopic osteochondral defect model [88]. It has also been demonstrated that osteocytes within autogenous bone fragments generated in situ during osteotomy procedures can reconnect with osteocytes in de novo bone found on the surface of such fragments, thereby restoring canalicular connectivity across the old–new bone interface [89]. In the lumbar vertebrae of toothed whale species *Physeter macrocephalus* and *Orcinus orca*, differences in Ot.LCN connectivity have been attributed to dissimilarities in metabolic activity and diving behaviour [90]. Furthermore, RCE has also found utility in detecting Ot.LCN in multimillion-year-old fossils (e.g., of a Late Cretaceous Mosasaur, Prognathodon) [91], examining the bacterial biofilm in MRONJ [92], and determining the extent of microdamage induced by self-tapping screw insertion in cadaver bone [93]. Finally, the combination of RCE and multibeam SEM has enabled the creation of seamlessly navigable anatomic maps of human bone and the relevant cellular components. The Google Maps JavaScript API navigational platform has been used to generate navigable maps of regions imaged using a 61-parallel-beam SEM (such as the Zeiss MultiSEM) [94]. Through a geographical information system approach, disease epidemiology can be conducted by network modelling, where spatial information is acquired by localising exposed landmarks such as osteocytes and blood vessels [95]. Introduction of machine learning to handle large datasets has further advanced the multibeam SEM approach. Knothe Tate et al. report a dataset comprising ~ 1810 mm^2^ tissue (> 7 million images and ~ 11 terrabytes of data) where ~ 206,180 osteocytes were detected and classified at > 92% accuracy within 100 h [96].

## Advantages of Resin Cast Etching and Comparison with Other Techniques

Although access to an SEM is necessary, RCE is an uncomplicated approach to direct visualisation of the Ot.LCN, even if moderately destructive. Specimen geometry is not a significant limitation. Large fields of view can be imaged quickly and the precise imaging conditions can be adjusted to achieve either improved contrast of resin-filled structures (e.g., at high accelerating voltage) or for finer details (e.g., low accelerating voltage and short working distance). Since no specific processing procedures such as demineralisation or contrast staining are necessary, RCE does not interfere with correlative analytical workflows [97]. Any clinical material (e.g., human bone biopsies) can be used provided that the specimen is undecalcified and embedded in resin. Within the SEM, the specimen may be tilted to high angles (up to 70° in some instruments) to reveal features hidden behind overhanging structures. Finally, a nominally intact surface can be easily recovered by polishing away the etched volume. It is important to note that, with RCE, the osteocyte cell body or the dendrites are not directly observed. What is seen, in fact, is the resin that has infiltrated into the pericellular space, enclosing the dendrite and the cell body. The lateral dimensions of the resin cast, therefore, represent the bony walls of the LCN. Therefore, the surface of the resin cast essentially reflects the inner wall of the Ot.LCN as opposed to the outer surface of the cell body or the dendrite. Besides RCE, several other analytical techniques can be used to image the Ot.LCN (Table 3).

**Table 3:** Other analytical techniques to image the osteocyte lacuno-canalicular network

Technique	Method	Advantage(s)	Disadvantage(s)
SEM	HCl–collagenase [98]		Dedicated specimen required
	EDTA–KOH [99]		Dedicated specimen required
	EDTA–NaOH [100]		Dedicated specimen required
	(P)FIB-SEM [101–103]	Serial sectioning and 3D reconstruction	Small volumes analysed; up to ~ 16,000 µm^3^ using PFIB [103]
	SBF-SEM [104]	Serial sectioning and 3D reconstruction Demineralisation (and OsO4 staining) improves contrast of subcellular structures and is less damaging to the ultramicrotome knife	Small volumes analysed. Metal implant (if present) must be removed. Demineralisation reduces contrast between cells and the surrounding matrix
TEM	Ultramicrotomy [24, 105]	Measurement of lateral dimensions of canaliculi, dendrites, microfilaments. Imaging of 0.5–1 µm thick sections using HVEM [108–110]. Tomography of 1–3 µm thick sections using UHVEM [113, 114]	Demineralisation is typically required. Metal implant (if present) must be removed [106–110]
	FIB lift-out [75, 111, 112]	Site-specific sample preparation Nanoscale characterisation of intact bone-biomaterial interface	Very small areas analysed (~ 20 × ~ 8 μm^2^)
CLSM	Fluorescent dyes [16, 36, 115–117]	Volumetric/depth imaging	Photobleaching
	Immunostaining [118–122]	Volumetric/depth imaging. Labelling of cytoskeleton, cell membrane, nucleus, gap junctions	Photobleaching
Multiphoton microscopy	3PEF [115, 125]	Intravital imaging of osteocytes [123]	Staining with fluorescent dyes Photobleaching
	THG [115, 125]	Label-free imaging of porosity (osteocytes, canaliculi, blood vessels)	Resin embedding reduces the contrast of canaliculi
Deconvolution microscopy	Fluorescent dyes [126]	Excellent lateral (x–y plane) resolution	Poor axial (z-direction) resolution. Need for fluorescent staining .
LSFM	Bone CLARITY [127]	3D fluorescence imaging up to 1.5 mm depth	Tissue clearing (including demineralisation and delipidation) required
SR-CT	X-rays [128–131]	High resolution and level of detail	Small volumes are sampled Limited access to SR facilities
AFM	EDTA [132] or HCl–NaOCl [133]	High resolution and level of detail Measurement of tissue mechanical properties	Surface preparation required to expose structures of interest Small areas are mapped

### Scanning Electron Microscopy

Various protocols have been described for stepwise removal of the inorganic and organic components of bone for direct observation of the cellular components using SEM. The HCl–collagenase method described by Ejiri and Ozawa [98] is analogous to the approaches involving ethylenediaminetetraacetic acid (EDTA) followed by either potassium hydroxide (KOH) [99] or sodium hydroxide (NaOH) [100] approaches. Paraformaldehyde and/or glutaraldehyde fixed bone specimens are used. In the first step, bone mineral is removed with HCl or EDTA, followed by elimination of the organic matrix by exposure to collagenase, KOH, or NaOH, and post-fixation with osmium tetroxide (OsO_4_). Although the bone specimen is not resin embedded, the 3D information obtained is remarkably similar to RCE. Two related SEM-based techniques generate 3D information through serial sectioning and 3D reconstruction, (*i*) focussed ion beam scanning electron microscopy (FIB-SEM) and (*ii*) serial block-face scanning electron microscopy (SBF-SEM).

FIB-SEM reveals significantly higher level of detail than the various optical imaging techniques. With gallium (Ga^3+^) as the liquid–metal ion source in most FIB instruments, serial sectioning is carried out for later 3D reconstruction. The main drawback of this approach is that extremely small volumes are typically analysed. Consequently, it is important to know beforehand where the osteocytes are located. Therefore, often, only those osteocytes are selected for imaging that are already exposed at the specimen surface (and therefore partially cut and incomplete) [101, 102]. This specific challenge is circumvented by recent advancements in plasma focussed ion beam (PFIB) technology, which enables large volume analysis (e.g., up to ~ 16,000 µm^3^) [103].

SBF-SEM involves automated, sequential BSE-SEM and ultramicrotome sectioning of a sample within the SEM. Both demineralised (decalcified and stained with OsO_4_) and undemineralised tissues can be used for SBF-SEM. The technique is, however, inherently destructive, the obtained images are non-isotropic, and the surface is rendered potentially unusable for correlative analytical platforms involving vibrational spectroscopy and crystallography [18, 104]. The advantages of decalcifying bone tissue for SBF-SEM include (*i*) greater ultrastructural image contrast, making automated segmentation of subcellular structures easier, (*ii*) improved image quality, and (*iii*) less damage to the diamond ultramicrotome knife. The disadvantages of decalcification are that (*i*) the tissue is further from the native state, (*ii*) the image contrast between the cells and surrounding matrix is reduced, making automatic segmentation of the lacunae more difficult, and (*iii*) the time required to prepare the decalcified sample is considerably longer than the mineralised sample [104].

### Transmission Electron Microscopy and Electron Tomography

Transmission electron microscopy (TEM) allows extremely precise measurement of lateral dimensions (i.e., diameter) of canaliculi, osteocyte dendrites (cytoplasmic processes), and microfilaments [105]. Routine TEM, using < 100 nm thick sections, also reveals artefacts related to sample preparation, e.g., shrinkage at the cell–matrix interface [24]. In the specific case of bone-implant specimens, if the TEM section must be obtained by ultramicrotomy, the metal implant must first be removed by either mechanical separation under a dissection microscope [106, 107] or cryofracturing [108–110]. Undemineralised, ultramicrotomed sections ranging in thickness from ~ 250–500 nm [108, 109] to 1 µm [110] have been examined using high voltage electron microscopy (HVEM) at 1000 kV. Using a FIB instrument to *lift-out* an electron transparent specimen, selected osteocytes (including those adjacent to metal implants) can be milled out from undemineralised bone for *Z*- (atomic number) contrast, scanning transmission electron microscopy [111, 112] and electron tomography [75]. Other possibilities for electron tomography include ultra-high voltage electron microscopy (UHVEM) at 2000 kV, where a ± 60° tilt series is collected at 2° intervals, using 1 µm thick sections [113], or 3 µm thick sections stained with silver proteinate (protargol) solution [114].

### Confocal Laser Scanning Microscopy

Several closely related methods using confocal laser scanning microscopy (CLSM) have been described for visualising the Ot.LCN. Ethanol fixed and dehydrated bone, stained with 0.1–1% fluorescein isothiocyanate (FITC) can be used for 2D [115] and 3D imaging [36, 116]. Similarly, formalin fixed and dehydrated bone can be stained with 1% Basic Fuchsin for determining lacunar volume, lacunar surface area, osteocyte density, and dendricity, however accurate measurement of volume and surface area of 3D structures requires an axial correction to overcome distortion in the *z*-direction [16]. Rhodamine-6G is another highly effective dye for imaging the LCN [117]. In avian/chicken bone, osteocytes can be selectively localised by incubation with the monoclonal antibody OB7.3, followed by fixation in 3% paraformaldehyde, permeabilisation with Triton-X, and incubation with an appropriate secondary antibody (e.g., Alexa Fluor® 488 conjugated chicken anti-mouse IgG antibody) [118]. Cell–cell communication across gap junctions between osteocytes can be observed by fluorescence recovery after photobleaching (FRAP) experiments [119, 120], where the gap junctions are localised by immunostaining for the protein connexin 43 [121]. More advanced *multiplexed* methods require demineralisation and immunostaining of the osteocyte cytoskeleton, cell membrane, nucleus, and the LCN, while the collagen can be visualised in transgenic mice expressing green fluorescent protein tagged collagen [122].

### Multiphoton Microscopy

Multiphoton microscopy, e.g., two- and three-photon excitation, enables intravital imaging of biological tissues such as bone [123]. Two-photon excited fluorescence (2PEF) and second harmonic generation (SHG) and primarily highlight the extracellular matrix [115, 124], while three-photon excited fluorescence (3PEF) and third harmonic generation (THG) reveal the Ot.LCN [115, 125]. In contrast to 2PEF and 3PEF, SHG and THG allow label-free imaging of certain fibrillar/tubular structures and heterogeneities such as interfaces within tissues.

### Deconvolution Microscopy

Deconvolution microscopy of formalin fixed and dehydrated bone, stained with 1% FITC also allows quantitative analysis of the LCN. A horizontal plane approximately 1 µm *above* the osteocyte cell body is imaged, which is expected to contain dendrites that fan out radially [126]. Despite excellent lateral resolution, radially extending dendrites cannot be assessed accurately owing to poor axial resolution.

### Light-Sheet Fluorescence Microscopy with Tissue Clearing

The tissue clearing method “Bone CLARITY” (Clear Lipid-exchanged Acrylamide-hybridised Rigid Imaging/Immunostaining/In situ hybridisation–compatible Tissue hydrogel) combined with light-sheet fluorescence microscopy (LSFM) enables visualisation of osteoprogenitors within bone marrow in whole/intact bone. Clearing renders intact bones transparent, for an imaging depth of up to ~ 1.5 mm, while preserving endogeneous fluorescence. Currently, this approach has only been reported for specialised, transgenic animal models, where fixation in 4% paraformaldehyde was followed by demineralisation in 10% EDTA demineralisation and delipidation with sodium dodecyl sulphate [127].

### Synchrotron Radiation X-ray Computed Tomography

Synchrotron radiation X-ray tomography (SR-CT) reveals the osteocytes and canalicular interconnectivity in exceptionally high detail. Although the scanned sub-volumes tend to be small [128–130], the obtained data on canalicular interconnectivity can be extended for simulation of LCN fluid flow dynamics [131].

### Atomic Force Microscopy

Imaging the LCN using atomic force microscopy (AFM) requires some form of surface preparation. A one-step demineralisation using EDTA is sufficient for unembedded bone [132] while a two-step procedure involving the use of HCl and NaOCl is required for poly(methyl methacrylate) embedded bone [133].

## Technical Challenges in Imaging Osteocytes Around Implant Biomaterials

Numerous challenges arise in the pursuit of visualising direct contact between implant biomaterials and the Ot.LCN within peri-implant bone. Techniques that require microtome sectioning (e.g., SBF-SEM) are not able to cut through certain materials, e.g., metals. As a result, mechanical removal of the metal from the bone-implant specimen, depending on the overall geometry and surface features, can unpredictably distort the integrity of the bone-implant interface zone. On the other hand, methods involving demineralisation (e.g., CLSM multiplexed imaging, bone CLARITY, SBF-SEM) pose the risk of distortion and displacement of metal implants and partial or complete elimination of acid-soluble CaP-based biomaterials. Although RCE does not require demineralisation or removal of a metal implant, minor technical difficulties may be experienced. For example, implant surfaces that are relatively smooth tend to afford poor mechanical interlocking between the implant and bone. Here, tissue distortion during sample preparation (i.e., shrinkage) allows for the development of a narrow gap at the bone-implant junction. This gap is subsequently filled by a thin layer of the embedding medium, which obscures the direct observation of osteocyte-implant contact using RCE [71, 72]. Canaliculi that had been in direct contact with the implant surface, while in situ, tend to appear separated from the implant surface. Certain experimental procedures also preclude direct visualisation of osteocyte attachment to the implant surface. Examples include various biomechanical tests to determine the strength of implant anchorage in bone, e.g., removal torque measurements [67], whereby the bone-implant interface is intentionally disrupted.

## In Conclusion

Although RCE has also found usefulness in comparing the Ot.LCN with matrix bound cells such as cementocytes (in cementum) [134] and odontoblasts (in dentine) [135], this technique has been most valuable in providing novel insights into the Ot.LCN of bone. More recently, correlative use of RCE and multiple other techniques such as fluorochrome labelling, TEM, BSE-SEM, histology, immunohistochemistry, polarised light microscopy, and SBF-SEM, has led to the emergence of a new hypothesis that osteocytes may be capable of depositing bone mineral into the pericellular space [136]. While backscattered electron imaging of polished bone specimens is typically not permissive of visualising canalicular connectivity (e.g., at the bone-implant junction) or discontinuity (e.g., at cement lines), RCE facilitates correlative/site-specific assessment [137], which can be easily combined with quantitative backscattered electron imaging (qBEI) and energy dispersive X-ray spectroscopy (EDX)—all within the scanning electron microscope. Despite the minor shortcomings, RCE provides sophisticated, quantitative and qualitative information about the Ot.LCN. With the potential for universal application to resin embedded bone, RCE deserves to be exploited further. Novel biomaterial compositions, including those that undergo in vivo degradation or possess controlled release capabilities, are expected to be an important focus area where RCE can help address questions related to osseointegration and bone-bonding ability of biomaterials.
